# Expression of Leukemia Inhibitory Factor (LIF) and p53 on Human Fetal Lung After Chorioamnionitis Suffering

**DOI:** 10.7759/cureus.76246

**Published:** 2024-12-23

**Authors:** Vasiliki M Kymioni, Konstantinos Kakleas, Lambropoulou Maria, Antonia Sioga, Kosmas Sarafidis, Sofia Karachrysafi, Theodore Lialiaris, Theodora Papamitsou

**Affiliations:** 1 Pediatrics, Agia Sofia Children's Hospital, Athens, GRC; 2 Allergy and Immunology, Agia Sofia Children's Hospital, Athens, GRC; 3 Histology-Embryology, Democritus University of Thrace, Alexandroupolis, GRC; 4 Histology-Embryology, Aristotle University of Thessaloniki, Thessaloniki, GRC; 5 1st Department of Neonatology and Neonatal Intensive Care, Ippokrateion General Hospital, Thessaloniki, GRC; 6 Department of Medicine, Faculty of Health Sciences, Aristotle University of Thessaloniki, Thessaloniki, GRC; 7 Laboratory of Histology-Embryology, Faculty of Health Sciences, Aristotle University of Thessaloniki, Thessaloniki, GRC; 8 Department of Ophthalmology, General Hospital “George Papanikolaou”, Thessaloniki, GRC; 9 Genetics, Democritus University of Thrace, Alexandroupolis, GRC

**Keywords:** chorioamnionitis, human fetal lung, immunochemistry, lif, p53

## Abstract

Introduction: Maternal infections such as chorioamnionitis could impact fetal lung development by altering cell proliferation and apoptosis. Chorioamnionitis favors the multiple pleiotropic cytokines production such as LIF (leukemia inhibitory factor) and an inflammation-related protein p53. The cytokine production can lead to lung tissue damage and lung disease development.

Aim: The aim of the study is to evaluate the expression and the interaction of pro-anti-inflammatory proteins LIF and p53 on fetal lungs of all developmental stages after chorioamnionitis exposure.

Methods: Lung tissue was obtained from stillborn fetuses through elective abortion or miscarriage and was examined. This immuno-histochemical study aimed to evaluate the expression of pro-anti-inflammatory proteins LIF, and p53 on fetal lung tissues exposed to chorioamnionitis during three different stages of fetal lung development. Moreover, the interaction between LIF and p53 on inflammatory lung tissue was evaluated.

Results: The comparison analysis of p53 rates in the three different stages showed that the p53 rate was significantly higher in the saccular stage compared to pseudogladular stage and canalicular stage, respectively (p-value: 0.05). The comparison analysis of LIF rates in the three different stages showed that the LIF rate was significantly higher in pseudogladular stage compared to the canalicular stage and saccular stage, respectively. No significant association between p53 and LIF was detected during the study duration period (p-value: 0.082).

Conclusion: The relationship between the two cytokines p53 and LIF interferes with the inflammation process. The result of their relation affects differently each stage of lung development as the cells maintain a different potential for differentiation and survival. Consequently, the extent of inflammation has a different importance at each stage.

## Introduction

Maternal infections such as chorioamnionitis could impact fetal lung development through amniotic fluid contact or placental-fetal circulation [1]. There is strong evidence that inflammation significantly alters cell proliferation and apoptosis, promoting lung disease in fetuses. When entire lung lobe segments from preterm children with acute respiratory distress syndrome were studied postmortem, considerable interstitial inflammation was also discovered throughout the lung tissue. However, it is still unclear when this process begins and how inflammatory reactions synced inside the uterus [2,3].

Chorioamnionitis favors multiple pleiotropic cytokines production such as LIF (leukemia inhibitory factor) which is ubiquitously expressed [4,5]. LIF belongs to the IL-6 inflammatory cytokines family expressing a pro- and anti-inflammatory function. Fetal development depends on LIF. In vitro, this protein is localized in multiple cells such as in syncytiotrophoblast, lung fibroblasts, endothelial cells, macrophages, monocytes, T-cells, thymus cells, neural cells, and mesangial cells. Its role is crucial in the implementation phase of trophoblast according to the results from mouse models [6-9]. LIF through multiple receptors with different final targets, contributes to the lung tissue development, maturation, and organization [7-9].

Another not well-studied but ubiquitous and inflammation-related protein is p53, which is a genome guardian and a reprogrammer of somatic cells. The functions of this protein are activation of DNA repairing proteins when its damage is serious but reversible and stabilization of cell cycle on G/S1 spot. This allows for repairing proteins to gain time for their action. When the damage is irreversible, apoptosis is activated. Finally, p53 is directly activated when telomeres are short in aged DNA [10,11].

Up to one-third of spontaneous abortions that occur during the second trimester of pregnancy are caused by chorioamnionitis [12]. The intriguing ambiguous relationship between chorioamnionitis and respiratory morbidity was initially demonstrated by Waterberg et al. [13]. Exposure to chorioamnionitis, which induces high levels of cytokines and other chemicals produced by inflammatory cells in the amniotic fluid, may have an impact on apoptosis and proliferation in the fetal lung. There have been reports of postnatally higher levels of neutrophils, macrophages, cytokines, and other mediators in the bronchoalveolar fluid of preterm infants with chronic lung disease of prematurity [14]. Until now, little is known about the development of the human lung under inflammatory circumstances. The exact role of p53 and LIF is not yet fully investigated.

This study aims to evaluate the expression of pro-anti-inflammatory proteins LIF and p53 on fetal lung tissues exposed to chorioamnionitis during three different stages of fetal lung development. Moreover, the interaction between LIF and p53 on inflammatory lung tissue will also be evaluated.

## Materials and methods

Lung tissue which was obtained from stillborn fetuses through elective abortion or miscarriage was examined. In all, 108 fetuses from all the trimesters of gestation were subcategorized into three groups according to the fetal lung development stage: the pseudoglandular stage (5-17 weeks of gestation), the canalicular stage (16-25 weeks of gestation), and the saccular stage (24 weeks to birth). Developmental criteria were used for the gestational age estimation. Sixty of the fetuses belonged to the pseudoglandular stage (5-15 weeks of gestation), 18 were in the canalicular stage (16-23 weeks of gestation), and 30 were in the saccular stage. The mothers of this group developed clinical signs of chorioamnionitis. All pregnancies were electively terminated. Demographic characteristics were the same for all mothers from whom the samples were taken, healthy, Caucasian, women with normal Body Mass Index (BMI). Fetuses with genetic diseases, and anatomical or systemic abnormalities were excluded as well as twin and multiple pregnancies. Paraffin-embedded specimens of 108 post-mortem human fetal lungs were obtained from the archives (2001-2014) of the Department of Histology-Embryology, Medical Faculty of Democritus University of Thrace, Alexandroupolis, Greece. Before the experiment, the approval of the Bioethics Committee of the Medical School of the Aristotle University of Thessaloniki was obtained.

All experiments were performed following all safety considerations and ethical guidelines were applied. The Bioethics and Ethics Committee of the Medical School of Aristotle University of Thessaloniki, after having reviewed the research protocol and the participant information and consent form at its meeting dated February 27, 2019, approved the conduct of the research (approval number 2.691).

Methods

Fetal lungs were cut as thick as five mm, then fixed in 10% neutral buffered formaldehyde at 4°C for 24 hours and processed for routine paraffin embedding. Four-micrometer-thick paraffin sections were cut from blocks containing sections from the lungs. The histological features of all fetuses were reassessed through sections stained with H&E (hematoxylin and eosin). Afterward, the paraffin blocks were used for immunohistochemical evaluation. 

Immunochemistry

The immunohistochemical protocol followed was similar to other studies [15,16]. Monoclonal antibodies against LIF and p53 were used and specimens were observed and evaluated under the BX40 binocular microscope (Olympus Corporation, Tokyo, Japan). The antibodies that were used were LIF (rabbit polyclonal ATLAS; Atlas Antibodies AB, Stockholm, Sweden) and p53 (monoclonal DO-7, DAKO; Agilent Technologies is located in: Santa Clara, California, USA).

Observation and statistics 

The intensity and extent of staining were evaluated using a semi-quantitative system. Every stained cell was scored as positive regardless of staining intensity. A range of quality values from 0 to 3 {negative (0), mild (1+), moderate (2+), and strong (3+)}, was determined as a scale for each of the parameters studied. The number of positive cells was counted through a 10×10 square calibrated grid inserted into the eyepiece of a BX40 binocular microscope (Olympus Corporation, Tokyo, Japan). Two independent researchers blindly observed the specimens without knowledge of the clinicopathological data.

The histologic diagnosis of chorioamnionitis was made on H&E-stained sections of the fetal membranes, following a standard protocol. The grade of inflammatory infiltrates by polymorphonuclear leukocytes (mild, moderate, and severe) at the level of the lesional tissue of the chorioamniotic plate was specifically taken into consideration.

Statistical analysis

Statistical analysis was performed using Statistical Package for the Social Sciences (SPSS), version 25.0 (IBM Corp., Armonk, NY, USA, released 2017). Chi-square tests were applied to assess distinctions among qualitative variables. Statistical significance was set at a p < 0.05 level.

## Results

A total of 108 samples were collected during the whole study period. Of the 108 samples, 60 were pseudogladular, 18 were canalicular and 30 were saccular. Of the 108 samples, 27/108 (25%) and 51/108 (47.2%) had p53 and LIF expression, respectively. Detailed data of the study population for each stage are presented in Table 1. 

**Table 1:** Comparison of LIF and p53 staining (negative, mild, moderate, strong) in each stage (pseudoglandular, canalicular, saccular). Statistically significant differences (p-value<0.05) are marked in bold. The result is significant at p < 0.05 (two-tailed hypothesis). LIF: leukemia inhibitory factor.

Group	Pseudoglandular stage		Canalicular stage		Saccular stage	
Antibodies/staining	LIF	p53	LIF	p53	LIF	p53
Negative (-) 0	30/60 (50%)	45/60 (75%)	9/18 (50%)	15/18 (83.3%)	18/30 (60%)	21/30 (70%)
Mild (+) 1	6/60 (10%)	6/60 (10%)	6/18 (33.3%)	0/18 (0)	3/30 (10%)	0/30 (0)
Moderate (++) 2	9/60 (15%)	3/60 (5%)	0/18 (0)	0/18 (0)	6/30 (20%)	0/30 (0)
Strong (+++) 3	15/60 (25%)	6/60 (10%)	3/18 (16.7%)	3/18 (16.7%)	3/30 (10%)	9/30 (10%)
Specimens' sum (100%)	60/60 (100%)	60/60 (100%)	18/18 (100%)	18/18 (100%)	30/30 (100%)	30/30 (100%)
p-values	<0.001		0.165		0.036	

**Figure 1:**
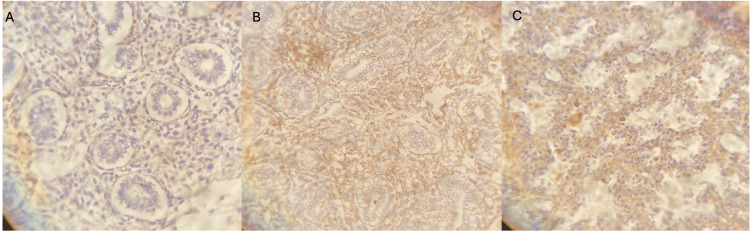
p53; different developmental stages and intensity staining (A) Tubular stage low intensity of p53 staining. (B) Pseudoglandular stage, medium intensity of p53 staining. (C) Alveolar sacs stage, low intensity of p53 staining

The comparison analysis of p53 rates in the three different stages showed that the p53 rate was significantly higher in the saccular stage compared to pseudogladular stage and canalicular stage, respectively {30% (9/30) in the saccular stage vs 10% (6/60) in pseudogladular stage and 16.7% (3/18) in canalicular stage (p-value: 0.05)}. In pseudoglandular stage, a huge percentage of specimens did not express p53 (75%). The rest of the specimens presented seriously lower percentages of p53 ranging from low to strong expression. In the other two stages of lung development, the experiment ended with the same result concerning the negative results. In the canalicular and saccular stages, only a strong expression of p53 {16,7% (3/18) and 10% (9/30)} was noticed, and no low or moderate expression. As a result, great disparities in our results were recorded (Table 1, Figure 1).

**Figure 2:**
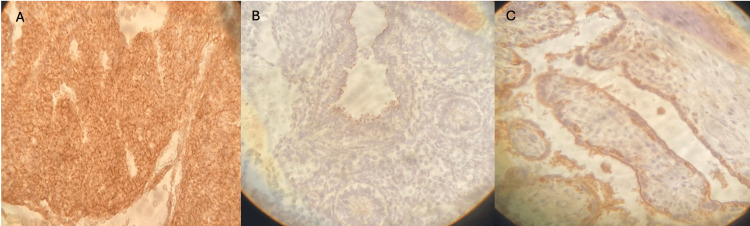
LIF; different developmental stages and intensity staining. (A) Pseudoglandular stage, strong intensity of LIF staining. (B) Pseudoglandular stage, low intensity of LIF staining only on big bronchus. (C) Tubular stage, medium intensity of LIF staining. LIF: leukemia inhibitory factor.

The comparison analysis of LIF rates in the three different stages showed that the LIF rate was significantly higher in pseudogladular stage compared to the canalicular stage and saccular stage, respectively {25% (15/60) in stage 1 vs 16.7% (3/18) in canalicular stage and 10% (3/30) in saccular stage, p-value: 0.05)}. In all three developmental stages, a significant number of specimens did not express LIF. Nevertheless, a LIF staining did not present the extreme expression ranges as noticed in the p53 case. In pseudoglandular stage, LIF had a strong expression (25%, 15/60). In the canalicular stage, LIF staining was mainly mild (33,3%, 6/18). Only 16,7% (3/18) of the specimens had a strong LIF expression. Finally, in the saccular stage, LIF was moderately detected (20%, 6/30). (Table 1, Figure 2).

No significant association between p53 and LIF was detected during the study duration period (p-value: 0.082). In the comparison analysis per stage, a significant negative association was detected between p53 and LIF in the pseudogladular stage (p-value<0.001) and in the saccular stage (p-value: 0.036), but not in the canalicular stage (p-value: 0.165).

## Discussion

In this study, we present a novel attempt to evaluate the detection of p53 and LIF in different stages of fetal lung development after exposure to chorioamnionitis. We have also tried to enlighten the possible relation between p53 and LIF. The results were extracted only through immunohistochemistry of human fetal lung tissue. Depicting the expression of these factors through pregnancy will help understand the impact of inflammation on human lung development.

LIF is not expressed in non-inflammatory tissues. Knight et al. support the idea of LIF mRNA expression in the normal cells of epithelial cells of the peripheral and central airways, mesenchymal cells, and nerve cells of the respiratory system [17]. Its action is autocrine or paracrine and participates in the decidua and placenta development. When binding to its receptor gp130, it promotes cell pleiotropism. On the other hand, binding on the LIF receptor (LIFr), leads to cell differentiation, and cell death [6]. In asthmatic children, LIF is localized in the respiratory tract smooth muscle cells, which substantiates its participation in the inflammatory pathways [18]. Animal studies have shown that deletion of the LIF gene fosters morphological changes in fetal lung development, especially in the type II pneumonocytes and the thickness of the alveolar-capillary membrane [19].

In the present study, LIF expression was higher in pseudoglandular stage, an early stage of lung development. Inflammation promotes the upregulation of LIF. Ulich et al. report that lipopolysaccharide (LPS) promotes inflammation in lung tissue when intratracheally injected, whereas on the contrary LIF downregulates inflammation downstream in mice airways [20]. LIF is moderately expressed in normal adult lung tissue on immunohistochemical staining according to the Human Protein Atlas [21]. Moreover, low expression of p53 in this stage, enhances LIF production, with the maintenance of cell pluripotency in early stages of development being the ultimate goal [22]. More evidence is required for the role of LIF in fetal development and neonatal and infantile lung diseases.

The tumor-suppressive properties of the p53 protein are important for normal cell cycle regulation and apoptosis during normal development [23]. In mouse models, it has been observed that p53 expression was increased in early gestation stages [23]. Its role is crucial in fetal morphogenesis and the development of the blood, neural, bone, fat, and muscle tissue from studies in animal models [14]. Upon completion of organogenesis, p53 was expressed to a lesser degree since the pluripotency of cells was eliminated [22,24],

Another important finding from this study is the reverse relation of LIF and p53, especially in the pseudoglandular stage where LIF is strongly expressed when p53 seems to be down-regulated. One possible explanation for this finding is that LIF could downregulate and degrade the expression of p53 via STAT3/MDM2 molecular pathway [5]. STAT3 binds on the promoter of p53 and suppresses its expression [25]. MDM2, an E3 ubiquitin ligase promotes p53 degradation through the proteasome [26]. In mouse models, MDM2 appears to be of high importance because of the postulation of apoptotic action of p53 [27]. On the other hand, both basal and inducible transcription of LIF can be regulated by p53 [28]. In murine and human cells, p53 through activation of the LIF pathway with WNT signaling, contributes to the maintenance of self-renewal and pluripotency [24]. On the other hand, LIF-induced inflammation control may result in impaired p53 expression and, consequently, suppression of apoptosis. In the saccular stage, p53 is more strongly expressed, as LIF is downregulated. Cells in this stage of development lose most of their pluripotency and the burden and duration of inflammation have more impact on cell survival. On the other hand, p53 expression was higher in the saccular stage, which is consistent with a more mature stage of fetal development. This finding can be explained by the maturity and the cell differentiation of this stage. Apoptosis and remodeling are more important for maintaining a proper tissue structure. Another explanation could be the more prolonged exposure to the inflammatory stimulus, inducing more damage and thus resulting in extensive apoptosis. 

In the canalicular stage, no significant difference between the expression of p53 and LIF was noticed. Inflammation and apoptosis are well-balanced during this stage. The maintenance of proliferation and differentiation capacity of cells is sustained as an important part of development [29]. The small number of samples collected in this group could have prevented the achievement of a statistically significant result. Another factor is that cells still maintain some of their pluripotency, therefore they can sustain and overcome easily the inflammatory stimuli. The duration and strength of the inflammation cannot be overlooked. 

There are several limitations of this study. First, the number of participants is small due to the low number of human specimens. Based on well-established and authorized techniques, the authors of the present study have tried to eliminate any potential small margin of error in the immunohistochemical evaluation of staining. The results of the present study are highly dependent on current staining methods, therefore data comparison with other studies that use different staining products should be interpreted cautiously. The primary difficulty in improving the reliability of results is the absence of an alternative laboratory technique.

## Conclusions

Human lung development is a dynamic course of action, influenced by the environment and supervised by the innate immune system of the fetus. The relationship between the cytokines p53 and LIF plays a critical role in regulating inflammation during lung development. Their interaction has distinct effects at different stages of lung maturation, as cells at each stage possess varying potentials for differentiation and survival. Consequently, the impact of inflammation differs in importance depending on the developmental stage. Understanding and modulating their expression could provide valuable insights into neonatal lung development and answer key questions about how inflammation influences this process. p53 in correlation with LIF, could predict with accuracy and certainty the possible lung disease development in the newborn. The value could be bigger for premature neonates. Further studies will be needed to examine their application in everyday medicine.
